# Venous Thrombosis in Acquired Hemophilia: The Complex Management of Competing Pathologies

**DOI:** 10.1055/s-0039-1698414

**Published:** 2019-10-09

**Authors:** Manu Chhabra, Zhen Wan Stephanie Hii, Joseph Rajendran, Kuperan Ponnudurai, Bingwen Eugene Fan

**Affiliations:** 1 Department of Hematology, Tan Tock Seng Hospital, Singapore, Singapore; 2 Khoo Teck Puat—National University Children's Medical Institute, National University Health System, Singapore, Singapore

**Keywords:** acquired hemophilia, deep vein thrombosis, thromboembolism

## Abstract

**Introduction** Venous thrombosis is rare in the setting of factor VIII (FVIII) deficiency. Cases of deep vein thrombosis (DVT) have been described in hemophiliacs after recent major surgery, or in association with the administration of FVIII concentrate and activated prothrombin complex concentrates, but occurrence of spontaneous DVT is even more uncommon.

**Aim** We describe the challenging management of extensive DVT in a patient with acquired hemophilia A with concurrent hemorrhagic manifestations and review similar published cases.

**Methods** We summarize a series of 10 cases with the following demographics: 6 males and 4 females; median age at presentation of 65 (21–80); mean inhibitor titer of 68.5 Bethesda Units (BU 1.9 to BU 350).

**Results** Four cases were idiopathic and six had associated conditions (cancer [two cases], recent pregnancy [two cases], and recent surgery [two cases]). Three cases had an inferior vena cava filter inserted for acute lower limb DVT/pulmonary embolism. Inhibitor eradication was achieved with high-dose steroids with or without cyclophosphamide, and adjunct Rituximab administration was used in three cases. One patient received concurrent therapeutic plasma exchange (TPE). Inhibitor eradication was fastest with concurrent TPE at 6 days (range: 6–733 days). The 30-day survival was 90%.

**Conclusions** There was adequate response of inhibitors to immunosuppression with steroids and cyclophosphamide therapy. For more refractory disease, Rituximab is emerging as a beneficial and cost-effective adjunct with better rates of complete remission, and the threshold for its use may be lowered in this complex cohort with dual competing pathologies.

## Introduction

Acquired hemophilia A is a rare hemorrhagic diathesis caused by the development of antibodies against factor VIII (FVIII). Over 50% of cases are idiopathic, and known associations include malignancy, an underlying autoimmune condition, and recent childbirth. Bleeding can be severe and confers a high morbidity and mortality of over 20%. 1 Treatment is with immunosuppression, and acute bleeding often necessitates securing hemostasis with FVIII concentrate, rFVIIa (recombinant factor VIIa), or activated prothrombin complex concentrates (aPCCs) such as FEIBA (factor VIII inhibitor bypass agent). 2

Venous thrombosis is rare in the setting of FVIII deficiency. 3 Cases of deep vein thrombosis (DVT) have been described in hemophiliacs after recent major surgery, or in association with the administration of FVIII concentrate and aPCCs, 4 but occurrence of spontaneous DVT is even more uncommon. 3 Here we present the paradoxical development and challenging management of extensive proximal lower limb DVT in a patient with simultaneous bleeding from acquired hemophilia A.

## Case Presentation

A 72-year-old male nonsmoker presented to the emergency department for a 1-month history of intermittent perirectal bleeding and progressive lower limb weakness. His medical history was of diabetes complicated by stage 4 chronic kidney disease (CKD), hypertension, and hyperlipidemia.

Examination revealed prominent bruising over the left flank, a hematoma over the right deltoid, and ecchymoses over the left inner arm, in conjunction with severe anemia (Hb 3.7 g/dL) and a prolonged activated partial thromboplastin time (APTT) (78.6 s), which was not fully correctible on 50% mixing studies (59 s). FVIII levels were low (<1%) and significant levels of FVIII inhibitor were detectable (82 Bethesda Units), establishing the diagnosis of acquired hemophilia A. Platelet and von Willebrand factor (VWF) levels were not deficient (480 × 10 ^9^ /L and >400%, respectively). A malignancy screen with computed tomography (CT) imaging and autoimmune markers were negative.

Additionally, the patient's left calf was noted to be swollen and tense and suspicion of a concurrent DVT was raised.

**Fig. 1:**
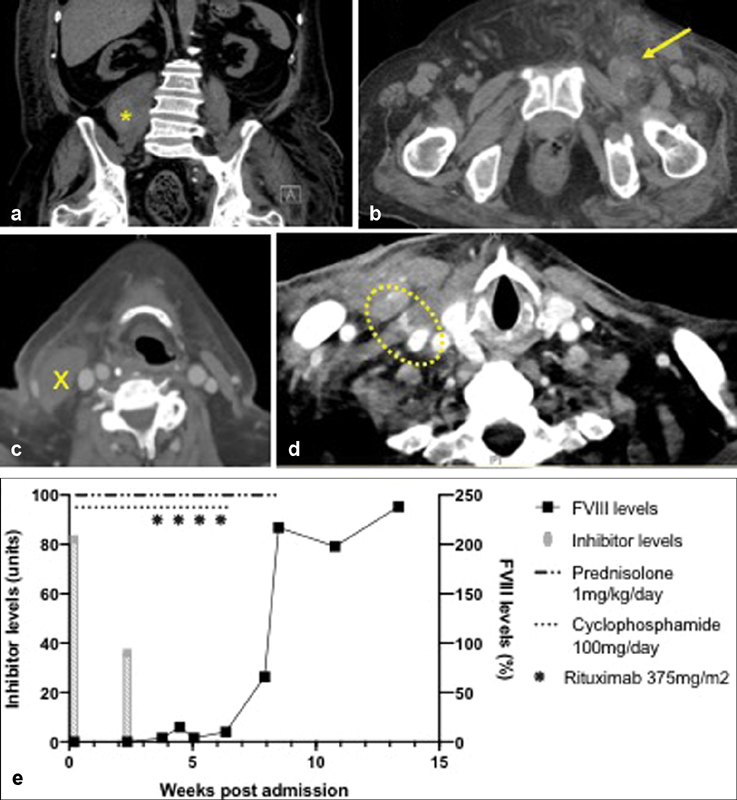
Simultaneous presentation of bleeding and thrombosis. ( **a, b** ) Noncontrast CT scan of abdomen and pelvis demonstrating ( **a** ) right psoas swelling, depicted with *star* , and ( **b** ) obliteration of the left femoral vein ( *arrow* ); ( **c, d** ) CT neck with contrast demonstrating ( **c** ) a hematoma overlying the right sternocleidomastoid ( *cross* ) and ( **d** ) contrast extravasation from the right IJV puncture site; ( **e** ) Serum FVIII and inhibitor levels with respect to immunosuppression therapy. CT, computed tomography; IJV, internal jugular vein.

An urgent abdominal ultrasound examination revealed a retroperitoneal collection, suggestive of a hematoma. A CT scan localized the hematoma to the right psoas muscle with significant subcutaneous flank edema, likely attributable to bleeding ( Fig. 1a ); the scan also detected significant thrombosis along the patient's left femoral vein ( Fig. 1b ). The patient was treated with 90 mcg/kg/dose of rFVIIa (NovoSeven), and immunosuppression with prednisolone (1 mg/kg/d) and cyclophosphamide (100 mg/d) was promptly commenced.

Extensive DVT involving the left common and superficial femoral veins and extending up to the left external and common iliac veins was subsequently confirmed with a Doppler ultrasound scan. Once acute bleeding had stabilized, inferior vena cava (IVC) filter insertion was performed on day 5 of admission, with periprocedural administration of rFVIIa. Due to technical difficulties, femoral venous access could not be secured, and the procedure was performed via the right internal jugular vein (IJV). The patient had mild oozing from the IJV insertion site and continued to receive rFVIIa till oozing had completely resolved. Approximately 24 hours after discontinuation of rFVIIa however, progressive bruising was noted over the IJV puncture site, and concerningly, the patient developed acute dysphonia. Urgent endoscopic examination discovered bruising and swelling over the right tongue base and aryepiglottic fold, suggesting extrinsic compression. Imaging revealed an expanding hematoma at the IJV puncture site with associated tracheal deviation ( Figs. 1c, d ). The patient was intubated expectantly and received regular rFVIIa doses, with monitoring in intensive care. Administration of rFVIIa was discontinued after 3 days when the neck bruising demonstrated clinical improvement and endoscopy confirmed resolution of airway compression, and extubation was successfully performed 24 hours later.

Despite reduction in inhibitor levels and slight recovery of FVIII levels with prednisolone and cyclophosphamide ( Fig. 1e ), the patient continued to have intermittent melena and developed a new trapezius hematoma with associated fall in serum hemoglobin, requiring packed cell and rescue rFVIIa administration. Endoscopy did not reveal any gastrointestinal lesions. He was commenced on second-line therapy with Rituximab (375 mg/m ^2^ per week in four doses), which resulted in gradual normalization of FVIII levels ( Fig. 1e ) and resolution of active hemorrhage.

At 8 weeks of therapy, prednisolone was tapered, and he was started on low-dose dose enoxaparin (40 mg/d; dose adjusted for stage III–IV renal impairment and increased risk of bleeding). Repeat Doppler ultrasonography of the left leg at 3 weeks following initiation of anticoagulation demonstrated complete resolution of the DVT. Enoxaparin was continued with the intention to complete 12 weeks of anticoagulation, or longer if the patient remained immobile, as immobility was identified as the primary prothrombotic risk factor. However, anticoagulation had to be discontinued after 8 weeks due to acute clinical deterioration and subsequent demise of the patient from a severe hospital-acquired pneumonia. Notably, there was no clinical recurrence of DVT or derangement of APTT values to suggest recurrence of his hemophilia.

## Discussion and Conclusions

We describe the rare development of extensive DVT in a patient with concurrent active hemorrhage from acquired hemophilia A, in the absence of significant risk factors for thrombosis.

DVT can be precipitated by recent surgery, pregnancy, malignancy, autoimmune disease, and inherited thrombophilia, 5 but in over half of cases no risk factors are identified, and other predisposing associations such as raised levels of FVIII and VWF may be contributory. 5 6 Our patient suffered from CKD, had several falls and poor mobility over the preceding weeks, and high VWF levels, which may have contributed to the development of DVT. 5 6 Without treatment, as many as half of proximal DVTs progress to cause pulmonary embolism; one in five of which is fatal. 7 Anticoagulation remains the cornerstone of therapy, and thrombolysis or thrombectomy can be considered for serious complications.

The pathophysiology of venous thrombosis is widely described as the product of stasis, hypercoagulability, and endovascular injury (Virchow's triad). Endothelial activation in response to endovascular injury releases VWF and P-selectin from Weibel–Palade bodies, triggering adhesion and activation of platelets, and recruitment of leucocytes and leukocyte microparticles containing tissue factor (TF). 8 TF can also be found in the perivascular tissue matrix exposed at sites of injury. 8 9 In the absence of vascular injury, the venous valvular sinuses have been proposed as a site for thrombus initiation, possibly as a consequence of relative venous stasis that lends to hypoxia, resulting in endothelial activation, and a procoagulant endothelial transcription state. 8 Exposure of TF and its association with factor VII (TF/FVIIa complex) convert small amounts of factor IX and X to their activated forms. In vitro studies suggest that the TF/FVIIa complex is rapidly degraded by TF pathway inhibitor (TFPI). 10

Propagation of the coagulation cascade requires that activated factor IX (FIXa) complex with its cofactor FVIII to generate the tenase complex that can catalyze activation of factor X via the intrinsic pathway. Lack of circulating FVIII would limit clot propagation via the intrinsic pathway and therefore, in the context of FVIII deficiency or inhibition, modulation of the extrinsic pathway by TFPI may be disproportionately important for thrombus initiation. 11 12 Generation of high levels of TF can overcome TFPI in vitro, 10 and could theoretically generate sufficient levels of TF/FVIIa complex to allow direct activation of factor X independently of the tenase complex, and propagate thrombus formation despite low FVIII levels. 13 14

We performed a literature review and identified nine cases of patients with acquired hemophilia A and concurrent thrombosis ( Table 1 ). 13 15 16 17 18 19 20 Most cases described the development of bleeding on the background of ongoing treatment for venous thrombosis. Several cases in the literature also discuss the difficulty of discriminating a DVT from an intramuscular hematoma or bleeding, with superimposed bruising and secondary extrinsic venous outflow obstruction. 13 16 Although typical Doppler ultrasound findings confer a high sensitivity and specificity for the diagnosis of venous thrombosis, discussion with a specialist about the need for further imaging may be appropriate in doubtful cases. 21

**Table 1:** Summary of published case reports on thrombosis in acquired hemophilia A

Study	Age/sex	Prothrombotic risk factors	Site of 1. Bleeding 2. Thrombosis	Relative timing	At presentation (of bleed)				Management of 1. Major Bleed 2. Inhibitor eradication 3. Thrombosis	FVIII b (%)	30-day survival
					APTT a (s)	Hb (g/dL)	FVIII (%)	Inhibitor (BU)			
Siow et al (1982) 15	70/M	Idiopathic	1. Right thigh hematoma, right knee hemarthrosis 2. Proximal right lower limb DVT	DVT first, with B12 deficiency	38 (13–38)	5.1, then 10	–	–	1. FFP 2. Steroids: hydrocortisone, prednisolone (4 wk) and cyclophosphamide (5 wk) 3. Anticoagulation (stopped after bleeding)	–	Yes
				Hematoma 18 days later on anticoagulation	120 (13–38)	8.5	–	Present			
Poli et al (1997) 16	36/F	Pregnancy (postpartum)	1. Ecchymoses 2. Distal right lower limb DVT c	Distal right DVT first	57 (on IV heparin)	–	–	–	1. Vitamin K 2. Prednisolone 1 g/kg/d (4 wk) 3. Heparin, then warfarin (stopped after bleeding)	Normal	Yes
				Ecchymoses on warfarin	110	–	1.9	1.9			
Deitcher et al (2002) 13	60/F	Idiopathic	1. Forearm hematoma 2. Right peroneal DVT	Simultaneous	54.3 (24–33)	–	5.3	57	1. FVIII concentrate and DDAVP; avoided aPCCs to reduce risk of propagating venous thrombosis 2. Not described 3. Anticoagulation for existing DVT stopped after bleeding manifested, IVC filter inserted; peroneal DVT not treated	149	Yes
	80/M	Lymphoma d	1. Left flank and thigh hematoma 2. Proximal left lower limb DVT	Simultaneous	61.4 (22–33)	–	2	47		353	Yes
	76/M	Postoperative	1. Large ecchymosis over trunk 2. Proximal left lower limb DVT	DVT first, bleeding while on warfarin	54.8 (22–33)	–	2	5		88	Yes
Spencer et al (2011) 17	37/F	Pregnancy (postpartum)	1. Bruising on upper limbs and right calf hematoma 2. Proximal right lower limb DVT	DVT first, with prolonged APTT (preheparin)	3.01 (0.88–1.16)	9.1	–	–	1. rFVIIa 2. Prednisolone 1 mg/kg/d (6 wk) and cyclophosphamide 100 mg/d (6 mo) 3. Heparin, then warfarin (stopped after hematoma)	62	Yes
				Bleeding 9 days later on warfarin	5.03 (0.88–1.16)	8.1	2	35			
Paudel et al (2016) 18	21/M	Abdominal trauma (gunshot wounds) and surgery	1. Gastrointestinal and mucosal bleeding, abdominal hematoma 2. Proximal right lower limb DVT, bilateral cephalic vein thrombi	DVT at day 14 postoperation	–	–	–	–	1. Vitamin K, FFP, rFVIIa 2. Methylprednisolone 120 mg/d, then Rituximab 375 mg/m ^2^ weekly (4 doses) 3. Heparin, then warfarin (stopped after bleeding)	–	Yes
				Major bleeding at day 24 on warfarin	>100	–	<1	12			
Wool et al (2017) 20	73/M	Idiopathic	1.Right thalamic hemorrhage with intraventricular extension 2. PE after rFVIIa	Right thalamic hemorrhage first, PE on day 5 rFVIIa therapy	76.9 (24–34)	–	4	27	1. FVIII concentrate (2 days), then rFVIIa (30 mcg/kg/dose) till plasma exchange (TPE) 2. Dexamethasone 8 mg BD; TPE day 5–6, then Rituximab 375 mg/m ^2^ weekly (2 doses) 3. IVC filter insertion	275	No e
Maral et al (2018) 19	46/F	Malignancy (adrenal)	1. Severe ecchymosis on extremities 2. IVC thrombosis (pre-existing)	Ecchymoses while on long-term warfarin for IVC thrombosis	76.9 (24–34)	–	3	350	1. Not discussed 2. Prednisolone 1 mg/kg/d and cyclophosphamide 500 mg/wk 3.Warfarin (stopped after ecchymoses)	Normal	Yes
Present case	72/M	Idiopathic	1. Right flank and right deltoid hematoma, right psoas hematoma, malena 2. Proximal left lower limb DVT	Simultaneous	79 (25–36)	3.7	<1	82	1. rFVIIa (90 mcg/kg/ dose) 2. Prednisolone 1 mg/kg/d (12 wk) and cyclophosphamide 100 mg/d (6 wk), Rituximab 375/m ^2^ (from day 26, 4 doses) 3. IVC filter, clexane	217	Yes

Abbreviations: aPCCs, activated prothrombin complex concentrates; APTT, activated partial thromboplastin time; BD, bis in die meaning twice a day; DDAVP, 1-desamino-8-d-arginine vasopressin/desmopressin; DVT, deep vein thrombosis; FVIII, human factor VII; FFP, fresh frozen plasma; Hb, hemoglobin; IVC, inferior vena cava; PE, plasma exchange; rFVIIa, recombinant activated FVII; TPE, therapeutic plasma exchange.

a Values in brackets represent control levels.

b Levels after completion of treatment.

c Interpreted as inappropriately diagnosed muscle hematoma.

d Splenic marginal zone lymphoma.

e Died on day 2s3 from arrhythmia.

All authors opted to prioritize the treatment of bleeding and suspend anticoagulation with the onset of hemorrhagic manifestations. Insertion of an IVC filter was used to reduce complications of pulmonary embolization from proximal lower limb DVT in some cases. 13 20 Access site bleeding during IVC filter insertion has an incidence of 6 to 15%, 22 but is likely to be more common and more severe in this patient group; the risks ought to be carefully discussed with the patient and performing proceduralist; choice of access site and periprocedure management also deserve careful consideration, 20 and prophylactic therapy with aPCCs and early investigation of any bleeding manifestations is prudent.

The use of FVIII concentrates and FVIII bypassing agents can promote propagation of existing venous thrombosis via both TF and TF-independent mechanisms. 23 24 Interestingly, unlike cases of venous thrombosis in congenital hemophilia whereby venous thrombosis was often associated with prior administration of FVIII concentrates or aPCCs, 4 occurrence of thrombosis in acquired hemophilia usually preceded administration of hemostatic agents, and no significant exacerbation of existing thrombus was reported after their administration, though notably, judicious use was reported by most authors. In cases with significant complications from thromboembolism, plasmapheresis was successfully used for rapid elimination of factor inhibitor to further minimize use of aPCCs. 20

In most of the cases reviewed, there was adequate response of inhibitors to immunosuppression with steroids and cyclophosphamide therapy. For more refractory disease, Rituximab is emerging as a beneficial and cost-effective adjunct with better rates of complete remission, 25 26 and the threshold for its use may be lowered in this complex cohort with dual competing pathologies.

## Ethics Approval

Data were obtained from the patient's existing case notes in accordance with the Personal Data Protection Act (2012) and the National Healthcare Group Personal Data Protection Policy. Being a single case prepared for publications, a formal DSRB approval was not required as per the institutional policy.

## Consent for Publication

All personal identifiers were removed following data collection.

The patient was deceased at the time of manuscript preparation. Formal consent was obtained from next of kin for use of the patient's clinical information and imaging for the purposes of research and education, as well as for paper and web publication.

## References

[1] Franchini M Lippi G Acquired factor VIII inhibitors Blood 2008 112 02 250 255 18463353 10.1182/blood-2008-03-143586

[2] Baudo F Collins P Huth-Kühne A Management of bleeding in acquired hemophilia A: results from the European Acquired Haemophilia (EACH2) Registry Blood 2012 120 01 39 46 22618709 10.1182/blood-2012-02-408930

[3] Martin K Key N S How I treat patients with inherited bleeding disorders who need anticoagulant therapy Blood 2016 128 02 178 184 27106121 10.1182/blood-2015-12-635094 PMC4946199

[4] Girolami A Scandellari R Zanon E Sartori R Girolami B Non-catheter associated venous thrombosis in hemophilia A and B. A critical review of all reported cases J Thromb Thrombolysis 2006 21 03 279 284 16683222 10.1007/s11239-006-6556-7

[5] Lijfering W M Rosendaal F R Cannegieter S C Risk factors for venous thrombosis - current understanding from an epidemiological point of view Br J Haematol 2010 149 06 824 833 20456358 10.1111/j.1365-2141.2010.08206.x

[6] Tsai A W Cushman M Rosamond W D Coagulation factors, inflammation markers, and venous thromboembolism: the longitudinal investigation of thromboembolism etiology (LITE) Am J Med 2002 113 08 636 642 12505113 10.1016/s0002-9343(02)01345-1

[7] Di Nisio M van Es N Büller H R Deep vein thrombosis and pulmonary embolism Lancet 2016 388(10063):3060 3073 27375038 10.1016/S0140-6736(16)30514-1

[8] Esmon C T Basic mechanisms and pathogenesis of venous thrombosis Blood Rev 2009 23 05 225 229 19683659 10.1016/j.blre.2009.07.002 PMC2762278

[9] Drake T A Morrissey J H Edgington T S Selective cellular expression of tissue factor in human tissues. Implications for disorders of hemostasis and thrombosis Am J Pathol 1989 134 05 1087 1097 2719077 PMC1879887

[10] van 't Veer C Hackeng T M Delahaye C Sixma J J Bouma B N Activated factor X and thrombin formation triggered by tissue factor on endothelial cell matrix in a flow model: effect of the tissue factor pathway inhibitor Blood 1994 84 04 1132 1142 8049429

[11] Nordfang O Valentin S Beck T C Hedner U Inhibition of extrinsic pathway inhibitor shortens the coagulation time of normal plasma and of hemophilia plasma Thromb Haemost 1991 66 04 464 467 1796397

[12] Erhardtsen E Ezban M Madsen M T Blocking of tissue factor pathway inhibitor (TFPI) shortens the bleeding time in rabbits with antibody induced haemophilia A Blood Coagul Fibrinolysis 1995 6 05 388 394 8589204 10.1097/00001721-199507000-00004

[13] Deitcher S R Carman T L Kottke-Marchant K Simultaneous deep venous thrombosis and acquired factor VIII inhibitor Clin Appl Thromb Hemost 2002 8 04 375 379 12516688 10.1177/107602960200800410

[14] Rao L V Rapaport S I Factor VIIa-catalyzed activation of factor X independent of tissue factor: its possible significance for control of hemophilic bleeding by infused factor VIIa Blood 1990 75 05 1069 1073 2306514

[15] Siow B L Nadarajah K Jayaratnam F J Acquired haemophilia. A case report Singapore Med J 1982 23 06 328 330 6820189

[16] Poli D Francois C Bini G Caciolli S Prisco D Acquired hemophilia mimicking deep vein thrombosis. Report of three cases Ann Ital Med Int 1997 12 166 168

[17] Spencer A Pearce M I Ames P R Sequential thrombosis and bleeding in a woman with a prolonged activated partial thromboplastin time Thromb J 2011 9 16 22032246 10.1186/1477-9560-9-16 PMC3213060

[18] Paudel R Dominguez L W Dogra P Suman S Badin S Wasserman C A hematological menace: multiple venous thrombosis complicated by acquired factor VIII deficiency Am J Case Rep 2016 17 214 218 27040655 10.12659/AJCR.895316 PMC4824342

[19] Maral S Bakanay S M Dilek I Acquired hemophilia with thrombosis in a cancer patient: an unusual presentation Blood Coagul Fibrinolysis 2018 29 01 129 130 29095762 10.1097/MBC.0000000000000670

[20] Wool G D Chapel D Treml A Miller J L Therapeutic plasma exchange as part of multimodal treatment of acquired hemophilia in a patient with concurrent acute intracerebral bleed and pulmonary embolism Transfusion 2017 57 07 1827 1832 28436106 10.1111/trf.14132

[21] Gaitini D Multimodality imaging of the peripheral venous system Int J Biomed Imaging 2007 2007 54616 18521181 10.1155/2007/54616 PMC1987337

[22] Joels C S Sing R F Heniford B T Complications of inferior vena cava filters Am Surg 2003 69 08 654 659 12953821

[23] Shibeko A M Woodle S A Lee T K Ovanesov M V Unifying the mechanism of recombinant FVIIa action: dose dependence is regulated differently by tissue factor and phospholipids Blood 2012 120 04 891 899 22563088 10.1182/blood-2011-11-393371 PMC3412350

[24] Augustsson C Persson E In vitro evidence of a tissue factor-independent mode of action of recombinant factor VIIa in hemophilia Blood 2014 124 20 3172 3174 25232061 10.1182/blood-2014-05-576892

[25] Kain S Copeland T S Leahy M F Treatment of refractory autoimmune (acquired) haemophilia with anti-CD20 (rituximab) Br J Haematol 2002 119 02 578 12406107 10.1046/j.1365-2141.2002.03835_5.x

[26] D'arena G Grandone E Di Minno M N Musto P Di Minno G The anti-CD20 monoclonal antibody rituximab to treat acquired haemophilia A Blood Transfus 2016 14 02 255 261 26509821 10.2450/2015.0090-15 PMC4918557

